# Social Media Intervention to Promote Smoking Treatment Utilization and Cessation Among Alaska Native People Who Smoke: Protocol for the Connecting Alaska Native People to Quit Smoking (CAN Quit) Pilot Study

**DOI:** 10.2196/15155

**Published:** 2019-11-22

**Authors:** Pamela S Sinicrope, Kathryn R Koller, Judith J Prochaska, Christine A Hughes, Martha J Bock, Paul A Decker, Christie A Flanagan, Zoe T Merritt, Crystal D Meade, Abbie L Willetto, Ken Resnicow, Timothy K Thomas, Christi A Patten

**Affiliations:** 1 Department of Psychiatry and Psychology and Behavioral Health Research Program Mayo Clinic Rochester, MN United States; 2 Clinical and Research Services Division of Community Health Services Alaska Native Tribal Health Consortium Anchorage, AK United States; 3 Stanford Prevention Research Center Department of Medicine Stanford University Stanford, CA United States; 4 Division of Biomedical Statistics and Informatics Department of Health Sciences Research Mayo Clinic Rochester, MN United States; 5 School of Public Health University of Michigan Ann Arbor, MI United States

**Keywords:** smoking, tobacco cessation, Alaska, Alaska Natives, tobacco smoking, internet, social media, clinical trial randomized, smoking cessation, intervention

## Abstract

**Background:**

Despite the high prevalence of tobacco use among Alaska Native (AN) people, tobacco cessation interventions developed specifically for this group are lacking. Social media hold promise as a scalable intervention strategy to promote smoking treatment utilization and cessation, given the barriers to treatment delivery (ie, geographic remoteness, limited funding, climate, and travel costs) in the state of Alaska (AK). Building on a longstanding tobacco control research partnership with the AK Tribal Health System, in this study, we are developing and pilot-testing a culturally relevant, Facebook (FB)-delivered intervention that incorporates a digital storytelling approach adapted from the effective Centers for Disease Control Tips from Former Smokers campaign.

**Objective:**

This study aims to promote evidence-based smoking treatment (eg, state quitline and Tribal cessation programs) uptake and cessation among AN people.

**Methods:**

This study fulfills the objectives for stage 1 of the National Institute on Drug Abuse behavioral integrative treatment development program. In stage 1a, we will use a mixed method approach to develop the FB intervention. Cultural variance and surface/deep structure frameworks will address the influence of culture in designing health messages. These developmental activities will include qualitative and quantitative assessments, followed by beta testing of proposed intervention content. In stage 1b, we will conduct a randomized pilot trial enrolling 60 AN adults who smoke. We will evaluate the feasibility, uptake, consumer response, and potential efficacy of the FB intervention compared with a control condition (quitline/treatment referral only). Primary outcome measures include feasibility and biochemically verified smoking abstinence at 1-, 3-, and 6-month follow-ups. Secondary outcomes will include self-reported smoking cessation treatment utilization and abstinence from tobacco/nicotine products. We will also explore interdependence (relationship orientation and collaborative efforts in lifestyle change) as a culturally relevant mediator of intervention efficacy.

**Results:**

The study enrolled 40 participants for phase 1, with data saturation being achieved at 30 AN people who smoke and 10 stakeholders. For phase 2, we enrolled 40 participants. Qualitative assessment of proposed intervention content was completed with 30 AN smokers and 10 stakeholders. We are currently analyzing data from the quantitative assessment with 40 participants in preparation for the beta testing, followed by the randomized pilot trial.

**Conclusions:**

The project is innovative for its use of social media communication tools that are culturally relevant in a behavioral intervention designed to reach AN people statewide to promote smoking treatment utilization and cessation. The study will further advance tobacco cessation research in an underserved disparity group. If the pilot intervention is successful, we will have a blueprint to conduct a large randomized controlled efficacy trial. Our approach could be considered for other remote AN communities to enhance the reach of evidence-based tobacco cessation treatments.

**International Registered Report Identifier (IRRID):**

DERR1-10.2196/15155

## Introduction

### Background and Significance

Cigarette smoking, the most preventable cause of morbidity, mortality, and excess health cost in the United States, accounts for 480,000 premature deaths yearly [1]. At 22%, American Indian (AI)/Alaska Native (AN) persons have the highest US smoking prevalence; and within this group, AN residents of Alaska (AK) have a prevalence of smoking more than double that of Alaskan whites (42% vs 17%) [2]. Accordingly, AN people who smoke experience more tobacco-related diseases and mortality compared with non-Native people living in AK or US whites [3-6]. A national public health objective is to reduce tobacco-caused health disparities [7,8]. The state of AK developed its own health improvement plan, Healthy Alaskans 2020, that identifies priority health indicators. Decreasing smoking prevalence among AN adults to 17% is one of the priorities [9]. We will address both objectives by developing effective strategies to decrease tobacco use within AN communities across the state of AK and among AN people as a whole. With approximately 20% of the state’s population self-identifying as AN race [10], substantial reductions in tobacco use will greatly contribute to reducing statewide tobacco use rates.

Quitlines have proven efficacy [11] but remain underutilized by AN people [12,13]. In addition, only 4% to 7% of unaided quit attempts are successful among AN smokers compared with 10% among non-Native smokers. Evidence-based counseling and medication treatments could boost quit rates to as high as 30% to 40% [14,15]. Therefore, a need exists to increase utilization of available, low/no cost, and evidence-based smoking cessation resources such as AK’s Tobacco Quitline or Tribal tobacco cessation resources.

The Alaska Native Tribal Health Consortium (ANTHC), a consortium of regional Tribal health leaders, represents diverse AN people in AK. ANTHC co-owns and comanages the Alaska Native Medical Center (ANMC) in Anchorage. ANMC provides specialty care to AN people statewide, serving as the AK Tribal Health System’s only tertiary care facility. ANTHC’s Community Health Services Tobacco Prevention and Control Program provides tobacco cessation counseling and nicotine replacement therapy (NRT) to inpatients at ANMC. Upon discharge, patients are referred to cessation services at their regional Tribal health organization, which vary widely, or to AK’s Tobacco Quitline. AK’s Tobacco Quitline, operated by the state, is available to all Alaskans but provides free NRT for only 1 month. Given these barriers, the ANTHC Tobacco Prevention and Control Program appealed to ANTHC’s Clinical and Research Services to explore ways to expand capacity for support of AN Tribal members’ cessation efforts statewide.

Although our study with AN pregnant women and youth demonstrated that face-to-face interventions had limited reach and efficacy [16,17], Web-based social networks, such as Facebook (FB), are potentially powerful tools for reaching, engaging, and connecting AN people who smoke in cessation efforts [18] because of their large reach, relatively low cost, and potential for greater adoption and sustainability. The following section reviews the literature on the topic of social media use for health promotion with ANs and, generally, in the area of tobacco cessation. Utilization of the internet to access health information and social media use have increased among AN people, even in remote regions. In a representative survey of 340 households in rural southwest AK (73% AN adults), 87% had at least 1 cell phone, 60% had a smartphone, and 81% used FB, Twitter, or other social media sites [19,20]. A survey of 362 AN females from a rural census area reported that 80% used internet, 78% had smartphones, and 90% used FB [21]. Although the potential of social media to enhance smoking cessation is understudied, it remains a priority research area [18,22,23], with some trials using social media platforms for smoking cessation reporting effectiveness [24-27].

An intervention with both a website and an FB group evaluated quasi-experimentally among 238 young adult smokers resulted in greater self-reported 7- and 30-day smoking abstinence rates and quit attempts, compared with an unmatched comparison group of quitline users at a 3-month follow-up [24]. Participants interacted significantly more with FB than the website; of FB users, 56% were men and 44% were women, with women more actively posting and engaging than men. An analysis of sex differences in communication styles revealed that women emphasized support and connecting, whereas men expressed strong assertions about quitting smoking [25].

Another study evaluated a 100-day Twitter intervention [26]. Among 160 smokers aged 18 to 59 years, Tweet2Quit doubled the rate of self-reported sustained abstinence at 2 months post quit date compared with a control condition (smokefree.gov cessation website referral plus nicotine patches), 40% versus 20%. Sex, but not age, was related to treatment outcome, with women less likely to quit smoking than men in both study conditions.

An uncontrolled FB study for young adult smokers tested whether monetary incentives enhanced FB intervention engagement [27]. FB engagement was high, did not differ by incentive, and self-reported smoking abstinence at 6 months was 18%. Another observational study evaluated social media to support smoking cessation efforts among participants enrolled in a state-run cessation program in Saudi Arabia. Investigators found that WhatsApp- and Twitter-based social media support groups were more likely to report a decrease in smoking frequency compared with those not using social media [27].

A total of 3 other feasibility studies examined FB use among smokers. Haines-Saah et al [28] found more postings to the *Picture Me Smokefree* FB page among women than men (ie, 189 total photos posted among women, mean 9.5, vs 94 posted by men, mean 4.2). Content analysis revealed that postings were of similar quality for both sexes; sharing of photos and captions about experiences with tobacco use and struggles with quitting in the context of family life and relationships were the main themes. Evaluation of the FB page smokefreewomen.gov [29] found that increased frequency of moderator postings to facilitate dialogue and provide support engaged existing and new users, resulting in a marked increase in user postings and reach. Finally, an FB intervention using health communication messaging and supportive moderator postings was associated with a decrease in cigarettes smoked per day from baseline to 2-week follow-up; increased engagement was associated with greater smoking reduction [30].

### Objective

To overcome barriers of geography, climate, and scalability, we proposed to create and pilot-test a culturally salient social media (ie, FB) intervention to promote evidence-based smoking treatment utilization and cessation for AN people that will eventually be maintained by ANTHC’s Tobacco Prevention and Control Program. The project builds on ANTHC and Mayo Clinic’s longstanding tobacco control research partnership with the AN community and is informed by our understanding of cultural factors that can both impede (eg, stress and adverse childhood experiences) and encourage (eg, close family ties and community values) cessation in this population. The goal of this formative study is to develop an FB intervention that will be a hidden and closed FB group. The goal of the randomized pilot trial is to obtain effect size estimates to adequately power a larger scale efficacy trial. For this phase of the research, the main outcome measures will be intervention feasibility (treatment acceptability and program satisfaction) and verified smoking abstinence. Secondary outcome measures will be self-reported other tobacco abstinence, smoking treatment utilization, and interdependence as a culturally relevant mediator of intervention effectiveness. Additional feasibility measures such as social media engagement, usability, and satisfaction will also be addressed.

The goal of the pilot trial is to obtain effect size estimates to adequately power a larger scale stage 2 efficacy trial.

### Theoretical Framework

**Figure 1:**
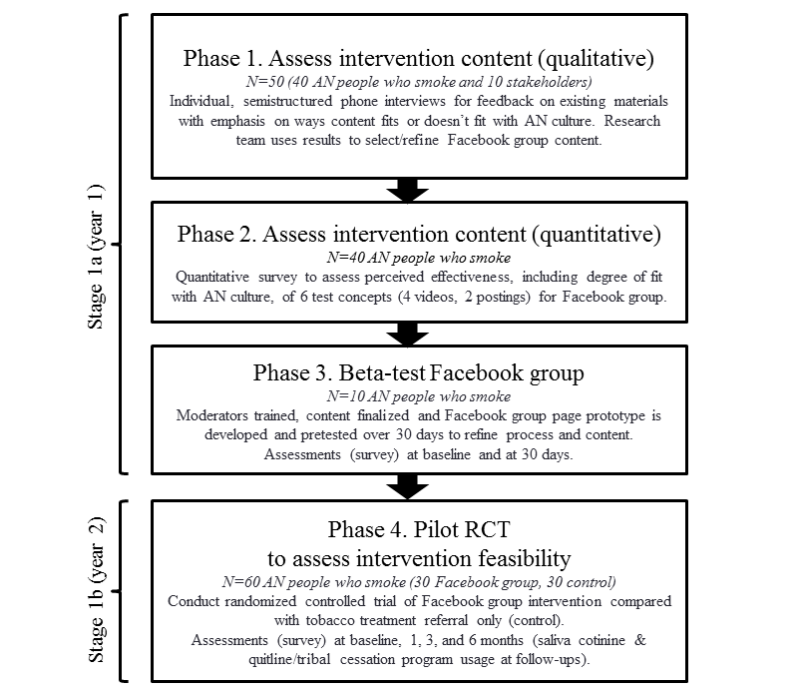
Overview of process to develop CAN Quit Facebook intervention. AN: Alaska Native; RCT: randomized controlled trial. CAN Quit: Connecting Alaska Native people to quit smoking (CAN Quit).

We used cultural variance and surface/deep structure frameworks [31,32] to address the influence of culture in designing health messages. Cultural variance framework considers AN cultural influences on health behaviors, including beliefs and norms (ie, communication styles and social acceptance of tobacco use), values (eg, interdependence), and AN knowledge systems/ways of knowing [33-38]. Surface and deep structure inform content and format of messages. Surface structure matches materials/messages to observable social and behavioral characteristics (eg, AN people, music, and clothing), and deep structure incorporates cultural beliefs and values. Surface structure generally enhances receptivity, comprehension, and acceptance of messages, whereas deep structure conveys salience. We will also use a planning framework based on the National Cancer Institute [39] and Centers for Disease Control (CDC) [40] recommendations for developing social media and other digital health communication tools, addressing key components of message construction [41,42] that are also consistent with stage 1 of the 3-stage model of behavioral therapies development [43] that includes intervention development, refinement, modification/adaptation, and pilot testing. In summary, we will conduct the research in 4 phases. In phase 1, we will use qualitative in-depth interviews and then in phase 2, quantitative methods to develop/refine message concepts. Next, in phase 3, we will develop and beta-test the intervention prototype; and finally, in phase 4, we will conduct a randomized pilot trial of the intervention (Figure 1).

### Intervention Content

Content for our FB intervention is culturally relevant, adapting the storytelling approach used in both the ANTHC digital stories about smoking cessation from AN people and the CDC Tips mass media campaign [44]. On the basis of factual health communication messaging, Tips features graphic, emotional, true stories from former smokers to increase awareness of smoking harms and encourage quitting. It includes the call to use free, evidence-based statewide quitline numbers and smoking cessation website resources (smokefree.gov). The campaign increased quitline utilization and quit attempts on a population level [44,45].

Tips stories promote salience and reduce tendency for smokers to discount adverse health outcomes as uncommon because stories feature real people [46]. Numerous studies, including a previous study by our team [33], suggest that storytelling is congruent with AN culture (strong oral storytelling tradition) [47-49], making the Tips format ideal for social media content development. Digital storytelling and other narrative forms of communication (eg, photonovela and photovoice) have emerged as important tools for health behavior change [50-52]. The communication forms reinforce traditional knowledge systems and cross-generational learning and build social connections [36,38]. Also relevant to the Tips campaign is the study conducted by our team indicating that AN adults preferred graphic, factual messages on tobacco use harms compared with other appeals, although this research was limited to interventions communicating risks during pregnancy [53,54].

The intervention will comprise an FB group moderated by a facilitator. To address potential concerns about FB privacy [55], we will utilize a closed and hidden FB group and a group policy/guideline that emphasizes confidentiality of all content. A hidden group is defined as invitation only, with the group and content not visible to anyone on FB except participants. Thus, anyone searching on FB would not find the group nor be able to request to join. Also, group membership or postings through news feeds will not be visible on the participants’ personal FB page.

We chose to use a moderated group based on research indicating that moderators play a critical role in directing and tailoring content to the group and enhancing overall social media engagement [25,56]. Also, the frequency of moderator FB postings is associated with increased participant engagement [29].

## Methods

### Overview

This study was reviewed and approved by the Institutional Review Boards for the AK area and Mayo Clinic. Tribal approval for the study was received from ANTHC. Our study fulfills objectives for stage 1 of the National Institute on Drug Abuse (NIDA) behavioral integrative treatment development program, where the intervention is developed in stage 1a and then evaluated for feasibility in 1b (Figure 1) [43].

### Participants and Recruitment

For all study phases, we will recruit AN men and women who smoke, statewide, using targeted and paid FB ads (ie, digital targeting) based on the following: (1) aged >19 years, (2) self-reported AN race/ethnicity, and (3) keywords related to tobacco use. FB ads are a successful method of recruiting for research studies, especially among hard-to-reach populations [57,58]. We developed ads that include an image and short text consistent with FB’s advertising guidelines. We will partner with organizations that have a large FB following, such as the Alaska Federation of Natives, along with community-specific FB pages to advertise the study. We will also advertise in Tribal newsletters, newspapers, and websites and hand out flyers.

Eligibility criteria for participation are as follows: (1) AN person (male or female); (2) aged >19 years; (3) smoked at least 1 cigarette per day over the past 7-day period; (4) person with cigarettes as main tobacco product used; (5) considering or willing to make a quit attempt; (6) has access to broadband (high speed) internet on a mobile phone, at home, work, or other location; (7) has an FB account or willing to set one up before study enrollment; and (8) has not been enrolled in a program or using pharmacotherapy to stop smoking over the last 3 months. Participants will participate in the study only once, not in multiple phases (Table 1).

**Table 1:** Participant eligibility and rationale for both the formative study and pilot trial.

Study inclusion criteria	Rationale
AN^a^ person (based on self-reported race/ethnicity) and resides in AK^b^	Study targets a population with the highest prevalence of tobacco use in the United States. We chose to conduct this initial study in AK to reduce sample and intervention design heterogeneity. Across the nation, there is immense cultural and geographic variability among ANs, for example, urban versus reservation dwelling and ceremonial versus nonceremonial tobacco use. ANs do not commonly use tobacco for ceremonial purposes. Also, AK has the highest percentage of AN residents versus all other states (19% vs 2%) [59,60]. If effective, the intervention could be adapted for and disseminated to AN adults nationwide.
Aged ≥19 years	Legal smoking age in AK is 19 years. Different social media venues and content may be warranted to address developmental issues among those <18 years. A Twitter-based intervention for adult smokers aged 20 to 59 years found that age was not related to engagement or cessation [61]; thus, we chose not to restrict the upper age limit.
Both men and women will be included	There are no preliminary data to indicate that sex-specific interventions are warranted at this stage of the research. We will explore sex differences on feasibility and efficacy as a research question.
Smoked at least 1 cigarette per day over the past 7-day period	This allows for participation of AN smokers who report fewer cigarettes per day and are considered *light* smokers but have cotinine concentrations equivalent to *heavy* white smokers, indicating differences in nicotine metabolism [15,62].
If other tobacco products are used, cigarettes are the main tobacco product used	Cigarette smoking in combination with other tobacco product use is highly prevalent in some AK rural regions [2]; thus, results are more generalizable if other tobacco use is allowed.
Considering or willing to make a quit attempt	Intervention promotes treatment utilization and quitting. We will explore readiness to quit as a potential moderator of FB engagement and efficacy.
Has access to broadband (high-speed) internet on mobile phone, at home, work, or other location	FB can be accessed on a variety of technology devices, such as computers, iPads, and mobile phones. Broadband internet access is needed to access social media and upload and download videos and other links.
Has an existing FB account or willing to set up an account before study enrollment	There is already good adoption of FB in rural regions of AK. Including participants familiar with regular social media interaction enhances participation, whereas nonusers or those unfamiliar with FB are less likely to engage in the intervention [61,63]. To provide study access to a broader group, we will offer a Web- or paper-based tutorial for those without an FB account.
For past 3 months, not enrolled in a program or using pharmacotherapy to stop smoking	Study promotes treatment uptake, utilization, and quitting.

^a^AN: Alaska Native.

^b^AK: Alaska.

Enrollment will occur online and by phone. All advertisements will contain the study toll-free phone number, email address, and a website link to Qualtrics, where interested participants can verify eligibility and enroll. Individuals emailing or calling will receive a brief description of the study and a link to the study website. The website will contain a short description of the study and eligibility criteria. Participants will receive a US $25 Visa gift card as appreciation for their participation for each assessment they complete.

### Measures

For all phases, we will ask the same sociodemographic and tobacco use questions: sex, age, Tribal affiliation, cultural identity (eg, language and traditionalism) [34], region of residence, marital status, education, employment, and current frequency of use of FB and other social media platforms. Tobacco use measures will include cigarettes per day, readiness to quit (Contemplation Ladder) [64], use of other tobacco and nicotine products, time to first cigarette after waking (<30 min vs >30 min) [65-67].

### Analyses

Sample characteristics will be summarized using descriptive statistics including means, percentages, and frequencies. Further analyses are described later by phase.

### Quality Assurance

For all phases, we use the same coordination and communication procedures successfully utilized in our previous study that include regular study team meetings held via teleconference and a systematic plan for following up with participants to ensure as high a follow-up rate as possible is achieved with regard to key outcomes. First, to track follow-up (number and type) and reasons for attrition, the study coordinator keeps a database of all contacted and consented participants. Generally, subjects are contacted up to about 3 times (with actual contact) before being considered lost to follow-up. This same process is followed for mailed saliva collection kits that include a self-addressed, stamped return envelope with every mailing. For survey data, missing data will be minimized through Web-based assessments using Qualtrics. An email link will be sent automatically by the Mayo Clinic Survey Research Center to complete the assessment. If a participant does not complete Web-based assessments, he/she will be contacted through email or by phone by the project coordinator and prompted to complete the assessment. The baseline and follow-up surveys will be done on the Web or by phone, or the survey will be mailed with a postage-paid return envelope depending on participant preference. These surveys will take about 15 to 30 min each to complete. Participants will be mailed a US $25 Visa gift card as remuneration for completing each assessment, including returned saliva cotinine kits (Mayo Clinic Laboratories).

### Stage 1a, Phases 1 to 3

We evaluated existing content using a mixed method approach, beginning with qualitative work to refine intervention content and a quantitative survey to evaluate the perceived effectiveness (PE) of selected content (phases 1-2). Once content was evaluated both qualitatively and quantitatively, a content library and moderator guide were developed, group moderators were trained, and the FB group prototype was developed. The complete FB group is now being beta-tested for final refinement (phase 3) using quantitative measures.

#### Stage 1a, Phase 1

##### Sample

We used a stratified purposeful sample [68] of AN adults who smoke with divisions based on audience segment (sex; age group 19-29, 30-49, and ≥50 years; and region—urban and rural). Krueger [69] recommends conducting at least 10 to 15 interviews per major subset before reaching data saturation, whereby no new information is being learned [69]. We estimate about 50 interviews, 40 with AN smokers (20 men, 20 women; 20 urban, 20 rural; 12-13 within each age group) and 10 with stakeholders (eg, AK’s Tobacco Quitline coaches and Tribal cessation program counselors) before reaching data saturation. An interview and moderator guide were developed to qualitatively assess potential intervention content. All participants being interviewed received a US $25 gift card for remuneration.

##### Procedures

###### Moderator Guide and Training

In all, 2 ANTHC research associates conducted interviews. They are experienced in phone interviewing and completed Tobacco Treatment Specialist training and training on qualitative research methods.

###### Analysis

Recordings were transcribed and content analysis [70] was performed using QSR NVivo software [71] to generate response themes. Codes and categories were developed based on moderator guide topics and themes emerging from the data. A total of 2 study team members coded responses for each topic area. During this open-coding process, themes were extracted for analysis when there was code endorsement or elaboration by several interviews. *In addition to open coding, planned comparisons within and across sex, age, and region strata were conducted and connections were made between identified categories.* Coding discrepancies were resolved through discussion with a third study team member until consensus was reached.

#### Stage 1a, Phase 2

From qualitative results, the research team selected 6 test concepts: 4 videos and 2 image/text moderator postings representing different types of appeals and message sources to evaluate for PE [72] via a quantitative Web-based survey.

##### Sample

We tested these concepts using a Web-based survey with a new sample of 40 AN adults who smoke (eligibility criteria Table 1) via a stratified purposeful sample [68], with divisions based on audience segment (eg, sex, age group, and urban/rural region) [69].

##### Procedures

Respondents viewed test concepts (eg, video, pictures, and text) embedded in a Web-based survey via Qualtrics survey software, or by phone if preferred, with the option to be mailed or emailed the concepts for review in advance of the survey.

###### Measures

Measures included a validated measure of PE to pretest each concept. PE is useful for assessing the likelihood of success of potential messages when large-scale efficacy pretesting for behavioral impact is impractical [72]. We used a 6-item validated measure of PE used to evaluate Tips stories [72], similar to PE measures used in other research [73]. After viewing each concept, respondents rated their level of agreement on a scale from 1 (strongly disagree) to 5 (strongly agree) with the following statements: (1) this was worth remembering; (2) this grabbed my attention; (3) this was powerful; (4) this was informative; (5) this was meaningful; and (6) this was convincing. Participants also rated each concept for *this fits with my culture*.

####### Analysis

PE items were summarized using descriptive statistics including means, percentages, and frequencies. The Chi-square goodness of fit test was used to summarize concepts most and least preferred by the participants. The associations of participant sex, age, and region with message concept preferences were examined using linear regression. Following the analysis of PE data, the research team reviewed and synthesized results from the previous phases to develop the prototype intervention for beta testing. Postings that were consistent with the content had high scores for PE, and those that qualitatively generated a positive reaction as being culturally salient and emotional and included images specific to AN culture as expressed by the interview participants were included.

#### Stage 1a, Phase 3

##### Prototype

The Mayo Clinic Social Media Department created the FB group page, and members of the research team developed the content library of moderator postings and set up the software to capture participant use data. Existing Tips and ANTHC digital stories deemed culturally acceptable in the formative phases were utilized for moderator postings. Additional content was added from an ANTHC photo library based on participant feedback and the expertise on AN culture provided by our ANTHC partners [47-49]. We created a content library of 66 postings that included 8 videos and 58 image/text postings. For each piece of content, a sample of accompanying text is provided for moderators to use, as well as a probe for generating further discussion or ways to respond to users’ questions. All sample text ends in a question to spark discussion among group members. All content includes the phone number of the AK state Quitline as well as a study-specific link that connects participants to the Quitline and AN Tribal cessation resources. Although the content library is crucial for generating discussion among participants, a central aspect of the intervention is the way that the moderators interact with group members. Therefore, in addition to rigorously refining our content, we also engaged our moderators in a structured training process where they were taught principles of group moderation, best practices for moderating and engaging participants, how to promote appropriate conversations and redirect engagement, and how to respond to difficult situations that often arise in a Web-based group among other topics. Moderators also participated in didactic training to learn about basic communication principles consistent with active listening and motivational interviewing. Finally, moderators were given the opportunity to practice their moderating skills with a social media expert/consultant using a role-playing format. This reinforced the content of the structured training and developed the moderators’ skills in writing posts to promote engagement, promoting participation and support of group members, and pulling in and welcoming new members. Moderators were encouraged to help generate daily participation in the FB group until group members became the main drivers of communication, but not to post new content daily, to avoid the risk of participants simply reading content rather than engaging with it.

The FB group is currently being beta-tested over a 30-day period. Results will be used to evaluate our processes and make any final refinements to our content (Figure 1).

##### Beta Test

###### Sample

We will beta-test the FB group with 10 AN adult smokers (Table 1) via a stratified purposeful sample [68], with divisions based on audience segment (eg, sex, age group, and urban/rural region) [69]. An FB group size of 10 was the minimum number for optimal engagement in previous studies [74,75]. The purpose of this phase will be to expose participants to the 30 days of moderator postings and obtain feedback to ensure that the system works as intended, note any technical issues that need to be remedied, and facilitate any refinements of the program.

###### Measures

We will utilize a Web-based survey that includes the 3-item Social Media Usability Measure: perceived ease of use, usefulness, and satisfaction rated on 5-point scales (1-strongly disagree and 5-strongly agree) [76] as well as open-ended questions. Refinements will be made based on user feedback.

###### Analysis

Sample characteristics, Social Media Usability measures, and concepts most and least preferred will be summarized using descriptive statistics including means, percentages, and frequencies. Open-ended questions will be summarized, and themes will be generated using content analysis [70]. Results from this phase are for beta testing purposes, only for purposes of refining the content for the pilot randomized trial.

#### Stage 1b, Phase 4

We will use rigorous design and methodology to evaluate the FB intervention’s feasibility and potential efficacy via a pilot randomized controlled trial.

##### Participants

Although not able to detect statistically significant study group differences on smoking abstinence, the study can obtain estimates of the intervention effect toward planning a definitive stage 2 efficacy trial. For the dichotomous variable of point prevalence abstinence, 30 subjects per condition should provide relatively stable group proportions for effect size estimates. Effect size estimates will include odds ratios for smoking abstinence. In addition to demonstrating feasibility, a doubling of the abstinence rate for the intervention versus control condition at 6 months will be considered to be of clinical significance and warrant proceeding to an efficacy trial [77]. This approach is consistent with recommendations for stage 1 study in behavioral addictions treatment development [43] and conducting small-scale trials to advance electronic health interventions [65]. Given the small sample size, proposed mediational analyses are exploratory.

Both recruitment and eligibility will be similar to the previous 3 phases of the research, whereby flyers and targeted FB ads of AN people who smoke will be used (Table 1).

##### Procedure

We will utilize a 2-arm, parallel-group, randomized controlled design with 60 participants randomized with 1:1 allocation to the intervention or control condition. Participants will be randomized within stratified blocks based on sex (ie, male or female), age group (eg, 19-29, 30-49, and ≥50 years), and region (ie, urban or rural)—potential variables related to outcomes [78,79]. Assessments will be conducted for both study groups at baseline and at 1-, 3-, and 6-month follow-ups. The primary outcomes are feasibility indicators and the 7-day biochemically confirmed smoking abstinence rate at the 6-month follow-up. Secondary end points are self-reported engagement in smoking cessation treatment and quit attempts (Figure 1).

##### Study Conditions

All participants will receive evidence-based [14] tobacco treatment referral information by postal mail (printed materials) and/or email, including information on their regional Tribal tobacco treatment program, state quitline and information on access to NRT, and smokefree.gov quit smoking resources. The control condition will receive no additional intervention provided by research staff. The intervention condition will, additionally, receive the FB intervention developed in stage 1a.

**Figure 2:**
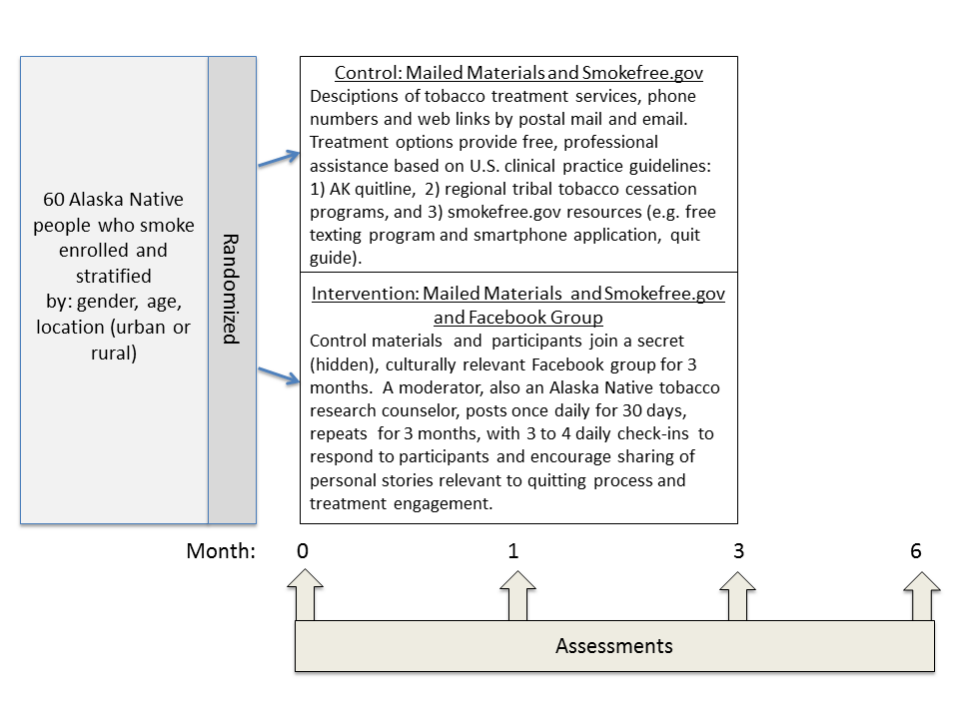
Study design. AK: Alaska; AN: Alaska Native.

The FB intervention, comprising 30 days of prewritten and evaluated postings, will be moderated daily by an ANTHC tobacco research counselor. When participants enter the study, they will be informed about the policies for posting content and that any inappropriate postings will be removed. As engagement may be optimal in the first 4 weeks, we will have 30 days of moderator postings available. However, we opted to have the FB group active for 3 months because participants might continue to engage in the intervention for continued social support; thus, the 30 postings will be repeated for each month the group is active. We will, therefore, measure engagement over time to empirically inform decisions about treatment duration in future trials. Accordingly, our assessments are timed to capture smoking behavior changes within the first 30 days (ie, 1 month), at the end of treatment (3 months), and at 6-month follow-up (Figure 2).

###### Feasibility

We will collect data on the number of potential participants screened, number eligible based on the inclusion/exclusion criteria, number of eligible participants enrolled, and reasons for exclusion or nonparticipation. The proportion of 60 participants completing the 6-month follow-up assessment (ie, retention) and the proportion providing a saliva cotinine specimen at each assessment will also be summarized. Treatment acceptability will be assessed with brief intervention satisfaction rating scales [16].

For each week of the study, we will extract for each intervention participant the following engagement metrics: number of logins; number of digital story downloads; number of user-generated posts, comments, questions, and responses to the moderator or other users; number of likes, shares, and reactions; and time and date of each. In addition, a transcript of all participant postings will be generated for content analysis.

###### Measures

Assessments will be completed at baseline and 1-, 3-, and 6-month follow-ups. With the exception of obtaining a saliva specimen from all participants for cotinine analysis, all measures will be administered on the Web using Qualtrics (Table 2).

**Table 2:** Pilot trial measures.

Measures	Baseline	1, 3, and 6 months
Sociodemographics and tobacco use	X	—^a^
Feasibility measures (eg, retention, Facebook use, and engagement)	—	X
Self-reported smoking abstinence	—	X
Self-reported tobacco/nicotine product use	X	X
Saliva cotinine to verify smoking abstinence	—	X
Self-reported smoking treatment utilization	—	X
Communal Orientation Scale (mediator)	X	X

^a^Not applicable.

###### Smoking Abstinence

At each follow-up, we will obtain self-reported cigarette use in the past 7 and 30 days, number of cigarettes smoked per day, and quit attempts. We will also assess current use of smokeless tobacco/*iqmik*, electronic cigarettes, and other tobacco/nicotine products. All participants will be mailed a saliva kit with a collection tube and postage-paid return envelope. Participants returning a saliva specimen will receive an additional US $25 Visa gift card. The specimen will be shipped to and assayed by Mayo Clinic laboratories, a standard approach for randomized trials, especially those with sample sizes <500 [30,79].

###### Smoking Treatment Utilization

As a secondary aim, we will document the self-reported use of any evidence-based cessation aid during the 6-month study period. For this pilot study, it is not practical to objectively verify self-reported treatment use given heterogeneity of potential services/medications used.

###### Communal Orientation Scale

The 14-item validated Communal Orientation Scale (COS) [80,81] will be administered at baseline and follow-up to examine interdependence as a culturally relevant mediator of intervention efficacy. In the intervention condition, we will also explore association of COS baseline scores with the degree of FB engagement, whereby a higher COS score would most likely be associated with a higher degree of engagement. This measure assesses the extent to which individuals are relationship- versus self-oriented.

#### Analysis

Recruitment data will be summarized, including the number of potential participants screened, number excluded for each inclusion/exclusion criteria, and number of eligible individuals agreeing to participate. To assess program reach, we will calculate proportion of subjects enrolled to total eligible subjects and compare enrollment rates by region (rural or urban) using the Chi-square test. Baseline demographics will be summarized and compared between study groups using the Chi-square test for categorical variables and the 2-sample *t* test/rank sum test for continuous variables. Percentage of enrolled participants completing each follow-up assessment (ie, study retention) and ratings of treatment acceptability will be compared between study groups using the Chi-square test (Fisher exact test). FB use and engagement will be summarized using descriptive statistics and time effects over the 3-month treatment period and assessed via mixed effects models as appropriate to explore sex, age group, and region effects, respectively. The association of COS baseline scores and FB engagement will be evaluated using linear regression. Qualitative (content) analysis [70] will be utilized to generate themes in FB postings and comments.

Biochemically confirmed 7-day point prevalence tobacco use rate at 1-, 3-, and 6-month follow-ups will be compared between conditions using logistic regression (with odds ratio and 95% confidence interval estimates). Using an intent-to-treat approach, we will classify participants eligible but lost to follow-up or not providing biochemical verification of smoking abstinence as smoking. We will also explore multiple imputation methods [82-84] to classify lost to follow-up as cigarette smokers or nonsmokers and conduct sensitivity analyses as appropriate. For these analyses, we will adjust for stratification factors (eg, sex, age group, and urban/rural region) and any baseline differences observed between treatment conditions if data allow (ie, adequate numbers of subjects verified as abstinent). Secondary analyses using logistic regression will explore intervention effects on self-reported abstinence from all tobacco/nicotine products, quit attempts, and self-reported tobacco treatment utilization. We will follow procedures suggested by MacKinnon [85,86] to assess mediation, fitting logistic/linear regression models to the data.

## Results

The study enrolled 40 participants for phase 1, with data saturation being achieved at 30 AN people who smoke and 10 stakeholders. For phase 2, we enrolled 40 participants. Qualitative assessment of proposed intervention content was completed with 30 AN smokers and 10 stakeholders. We are currently analyzing data from the quantitative assessment with 40 participants in preparation for the beta testing, followed by the randomized pilot trial.

## Discussion

### Principal Findings

This multistage pilot project will develop a social media intervention to promote smoking cessation among AN people through utilization of existing evidence-based approaches, such as AK’s Tobacco Quitline.

The proposed study, focusing on AN smokers, advances the methods of published social media intervention studies through the use of biochemical verification of smoking abstinence and extended duration of follow-up. Previously, most studies have targeted only young adults, whereas we plan to include a wide age range. We will also explore potential sex, age, and regional (urban/rural) effects on FB engagement and quitting, as there is limited research exploring these variables within the context of social media platforms for smoking cessation [24,26]. Within smoking cessation intervention efficacy and effectiveness trials generally, a recent literature review on sex/gender differences found that of 126 tests conducted, only 2 observed that women were significantly more likely to quit smoking than men, compared with 59 that found women were significantly less likely to quit smoking than men; the remaining 65 studies reported no difference by gender [87].

### Strengths

This pilot project is innovative for using social media communication tools that are culturally relevant and have already been adopted and that create statewide intervention access, thus promoting a scalable and sustainable approach that is tailored to the culture of AN people. The study is significant because it will advance research on population-specific treatments for ANs, an underserved, tobacco-use disparity group. If the pilot intervention is successful, we will have a blueprint to conduct a large randomized controlled efficacy trial.

### Limitations

Despite our strong mixed method experimental design, there are some limitations to our approach. First of all, although FB adoption is high among AN people overall, those not using social media will not be reached by this intervention. It is possible that some age groups will be more represented in this study than others because of possible gender-based differences in FB use and engagement. Finally, from the study design, we will not be able to assess the relative contribution of each component to intervention efficacy. Furthermore, FB utilizes certain algorithms to notify their users about topics that may be of interest to them based on their usage. However, as this is a hidden and closed group, none of the page posts will be added to a user’s news feed. Despite this, it is possible that exposure to Quitline-related ads or posts may be increased among those who participate in the group—an aspect to an FB intervention that the authors will have no control over. To explore these possible exposures, we will query intervention participants at the close of the FB group, a question about the perceptions about whether or not they received more than normal notifications, posts, or ads related to smoking, smoking cessation, or quitlines.

### Conclusions

The described intervention has potential for promoting engagement with evidence-based smoking cessation treatment including AK’s Tobacco Quitline and Tribal cessation programs statewide and holds promise for AN people because it is scalable and sustainable. It utilizes a popular channel of communication and an existing, evidence-based treatment that could be considered for other remote AN communities to enhance the reach of evidence-based tobacco cessation treatments.

## Appendix

Multimedia Appendix 1 Peer-review reports.

## Abbreviations

AI: American Indian
AK: Alaska
AN: Alaska Native
ANMC: Alaska Native Medical Center
ANTHC: The Alaska Native Tribal Health Consortium
CDC: Centers for Disease Control
COS: Communal Orientation Scale
FB: Facebook
NIDA: National Institute on Drug Abuse
NRT: nicotine replacement therapy
PE: perceived effectiveness
