# Supplementary material for: Social Media Intervention to Promote Smoking Treatment Utilization and Cessation Among Alaska Native People Who Smoke: Protocol for the Connecting Alaska Native People to Quit Smoking (CAN Quit) Pilot Study
Source: JMIR Res Protoc. 2019 Nov 22;8(11):e15155. doi: 10.2196/15155 (PMC6898890; doi:10.2196/15155)
Supplement: Multimedia Appendix 1 [file resprot_v8i11e15155_app1.pdf]

SUMMARY STATEMENT

(Privileged Communication)

PROGRAM CONTACT:
Will Aklin
301-443-3207
aklinwm@mail.nih.gov

Release Date: **10/23/2017**
Revised Date:

Application Number: **1 R34 DA046008-01**

Principal Investigator

PATTEN, CHRISTI A

Applicant Organization: MAYO CLINIC ROCHESTER

Review Group: **IPTA**
Interventions to Prevent and Treat Addictions Study Section

Meeting Date: **10/12/2017**
Council: **JAN 2018**
Requested Start: **04/01/2018**
RFA/PA: **PA16-073**
PCC: **CC/WMA**

Project Title: **Social Media Intervention to Promote Smoking Treatment Utilization and Cessation among Alaska Native Smokers**
SRG Action: **Impact Score:30**
Next Steps: **Visit https://grants.nih.gov/grants/next_steps.htm**
Human Subjects: **30-Human subjects involved - Certified, no SRG concerns**
Animal Subjects: **10-No live vertebrate animals involved for competing appl.**
Gender: **1A-Both genders, scientifically acceptable**
Minority: **2A-Only minorities, scientifically acceptable**
Children: **3A-No children included, scientifically acceptable**
Clinical Research - not NIH-defined Phase III Trial

Project Year	Direct Costs Requested	Estimated Total Cost
1	150,000	212,410
2	150,000	212,410
3	150,000	212,410
TOTAL	450,000	637,230

ADMINISTRATIVE BUDGET NOTE: The budget shown is the requested budget and has not been adjusted to reflect any recommendations made by reviewers. If an award is planned, the costs will be calculated by Institute grants management staff based on the recommendations outlined below in the COMMITTEE BUDGET RECOMMENDATIONS section.

1R34DA046008-01 Patten, Christi

RESUME AND SUMMARY OF DISCUSSION: This application proposes to develop and pilot-test a culturally relevant, Facebook-delivered intervention to promote smoking treatment uptake and cessation among Alaska Native (AN) smokers. The significance of targeting a scalable, culturally relevant social media intervention to this underserved population with the highest prevalence of tobacco use among racial/ethnic groups, and low rates of treatment utilization, is very high. During the discussion, reviewers were enthusiastic about the significance of the high priority disparity population, the exceptional investigators and environment with longstanding tobacco control research partnership with the AN community, and the high scientific rigor including biochemical verification of smoking abstinence and well-described intervention development procedures. While the scientific premise is generally strong and supported by a balanced review of high-quality research, it is weakened by the lack of evidence provided that AN respond to similar interventions by seeking formal help to quit smoking, and that AN smokers need culturally-tailored interventions. Minor weaknesses were also identified in the approach, including that quitline treatment utilization is measured only by self-report, health literacy is not addressed, the proposed intervention relies on potentially less effective loss-framed messages, and there is a lack of clarity regarding participant flow rates and whether rolling admission will be used. Overall, however, the strengths of this highly significant application from an outstanding team outweigh these weaknesses, and the proposed project has the potential for high impact.

DESCRIPTION (provided by applicant): Nationally, the prevalence of tobacco use is highest among Alaska Native (AN) people and tobacco cessation interventions developed specifically for this disparity group are lacking. Geographic remoteness, climate, and travel costs are key barriers to treatment delivery. Social media has promise as a scalable intervention strategy to promote smoking treatment utilization and cessation for AN smokers. Building on our team's longstanding tobacco control research partnership with the AN community, we propose to develop and pilot-test a culturally relevant, Facebook-delivered intervention to promote smoking treatment uptake and cessation among AN smokers. The Facebook content will include a digital storytelling approach adapted from the effective CDC Tips™ from Former Smokers campaign. The Facebook intervention will promote the use of evidence-based treatment, e.g., state quitline and tribal cessation programs. This R34 application is submitted in response to PA-16-073, fulfilling the objectives for Stage I of the NIDA behavioral integrative treatment development program. In Stage Ia, we will develop the Facebook intervention. This formative research will use the cultural variance and surface/deep structure frameworks to address the influence of culture in designing health messages, and adopt qualitative and quantitative pretesting methods to develop and beta-test the intervention prototype. In Stage Ib, we will conduct a randomized pilot trial enrolling 60 adult AN smokers to evaluate the feasibility, uptake, consumer response, and potential efficacy of the Facebook intervention, compared to a control condition (quitline/treatment referral). The primary outcomes will be feasibility (e.g., Facebook engagement) and the biochemically-verified smoking abstinence rate at 1, 3, and 6 months follow-up. Secondary outcomes will include self-reported smoking cessation treatment utilization and abstinence from all tobacco/nicotine products. We will also explore interdependence (relationship orientation and collaborative efforts in lifestyle change) as a culturally relevant mediator of intervention efficacy. A community advisory committee will guide all project activities. The project is innovative for developing a new behavioral intervention to reach AN people statewide to promote smoking treatment utilization and cessation using social media communication tools that are culturally relevant and have already been adopted. The study is significant because it will advance research on population-specific treatments for an underserved, AN, tobacco-use disparity group. If the pilot intervention is successful, we will have a blueprint to conduct a large, randomized, controlled, efficacy trial.

PUBLIC HEALTH RELEVANCE: The highest rate of tobacco use in the US is among Alaska Native (AN) people; however, there is a lack of interventions for tobacco cessation developed specifically for

this group. Social media is a potential way to reach AN smokers statewide to engage them in smoking cessation treatment and to promote smoking cessation. This project builds on our longstanding partnership with the AN community to develop and evaluate a social media (Facebook) intervention to promote smoking treatment use and cessation among AN smokers.

CRITIQUE 1

Significance: 4
Investigator(s): 1
Innovation: 2
Approach: 4
Environment: 1

Overall Impact: This is a novel and significant application that proposes to evaluate the feasibility of a Facebook group for Alaska Native (AN) smokers. The study is strengthened by already-tested recruitment methods, thorough rationale of inclusion criteria, partnership with key stakeholders and a community advisory board, collection of biochemical data, and intervention development framework. Also, the proposed intervention draws on the strengths of an already-established CDC intervention that included a Facebook event. The application had few weaknesses including the need for a stronger premise that promoting engagement would be associated with utilization and behavioral change in this population; more clarification whether assignment to the intervention would be on a rolling basis; and clarity on participant flow rates. Overall, this study is likely to have a big impact if it were successful and has the potential to reach a large population at the state-level who may not otherwise access evidence-based treatments.

1. Significance:

Strengths

- Strong premise that builds off face-to-face smoking cessation interventions with AN pregnant women and youth that have had limited reach and efficacy; acknowledges web-interventions are associated with very low utilization and suggests interactive social media interventions as a solution. Based on previous data and studies that most ANs use Facebook and successful recruitment of AN tobacco users for research. Good review of the previous trials that have done social media interventions – preliminary focus groups of AN tobacco users revealed that participants had highest ratings for videos featuring AN people justifying the cultural adaptation.
- Lack of tobacco cessation interventions for American Indians and Alaska Native (AI/AN) persons.
- If aims are achieved, there would be significant impact as the intervention could be disseminated widely for a disparity group through social media for this vulnerable group.

Weaknesses

- No discussion of proportion of ANs in the existing social media and feasibility trials.
- Prior research from the investigators note that previous face-to-face trials have limited efficacy, how will the proposed lower-intensity intervention be different in content aside from the digital storytelling? More justification for the proposed intervention’s effect on smoking would strengthen premise (e.g., any evidence to suggest that increased exposure to quit messages and treatments will increase AN utilization in smoking or other target behaviors?).

2. Investigator(s):

Strengths

- Dr. Patten has worked with the AN community for the past 16 years to reduce tobacco use.
- Longstanding tobacco control research partnership with AN community.
- Successful partnership between Mayo Clinic and ANTHC for the past 16 years.
- Several experts working in tobacco cessation with rural AN including Drs. Resnicow and Prochaska.
- Partnership with CDC Health Communications Branch who developed the intervention that will be adapted (no support letter, but email indicating unofficial commitment to provide technical assistance).

Weaknesses

- None noted.

3. Innovation:

Strengths

- Social media intervention culturally tailored to AN people, who tend not to utilize quitlines, but are on social media.
- State-wide intervention potential.
- Focus on AN smokers is innovative including cultural adaptation using digital storytelling.

Weaknesses

- Online and social media interventions are not particularly innovative.

4. Approach:

Strengths

- Biochemical data collection.
- Prior successful recruitment of AN tobacco users for research.
- Highlights sex differences in previous trials and gender effects that women may be less likely to quit.
- Intervention based on storytelling from CDC Tips' mass media campaign, which has increased quitline utilization and quit attempts at a population-level.
- Storytelling and narrative forms of communication are especially effective for engagement for AN people with a strong oral tradition (unclear regarding outcomes).
- Community Advisory Board of 10 members to guide all project activities.
- Thorough inclusion criteria and rationale.
- Intervention based on prior content from CDC Tips and a CDC Facebook event where a moderator posted once a day (identical to what is proposed).
- Takes into account possibility that federal funding for AK quitline services might diminish and offers alternatives (e.g., smokefree.gov).
- Thorough intervention development proposed including use of a health communication framework; 40 interviews during qualitative pre-testing.

Weaknesses

- When joining a Facebook group, typically first and last names will be shown and eligible individuals should be made aware during the consent process that their identity will be revealed.
- It is unclear whether there will be rolling admission to the Facebook intervention or if all intervention participants will start at the same time. If rolling, how will this be controlled for in the analyses and what were the weaknesses of their previous trials?
- Can a wide age range affect participant self-disclosure in the Facebook group? Would younger participants, for example, be less likely to participate if older participants are present?
- Examples of moderator postings would be helpful. How are they different from existing intervention content by the investigators that have limited efficacy?
- Unclear flow rates – how long will the recruitment period be for?
- How much per participant will be allocated to Facebook advertisements?
- Unclear if they have CDC's permission to adapt the intervention.

5. Environment:

Strengths

- Mayo Clinic is a stellar place to carry out the research and their long-standing research collaborative with ANTHC is a strength.

Weaknesses

- None noted.

Protections for Human Subjects:

Acceptable Risks and/or Adequate Protections

- Protections for human subjects are adequate and well described; benefits offset the risks.

Data and Safety Monitoring Plan (Applicable for Clinical Trials Only):

Acceptable

- The DSMP adequately addresses data management and participant safety concerns.

Inclusion of Women, Minorities and Children:

- Sex/Gender: Distribution justified scientifically
- Race/Ethnicity: Distribution justified scientifically
- For NIH-Defined Phase III trials, Plans for valid design and analysis: Not applicable
- Inclusion/Exclusion of Children under 18: Excluding ages <18; justified scientifically

Vertebrate Animals:

Not Applicable (No Vertebrate Animals)

Biohazards:

Acceptable

- Adequate protection proposed.

Resource Sharing Plans:

Acceptable

Budget and Period of Support:

Recommend as Requested

CRITIQUE 2

Significance: 4
Investigator(s): 1
Innovation: 1
Approach: 4
Environment: 1

Overall Impact: This innovative R34 proposes to develop and test a social media intervention on Facebook to promote smoking cessation among Alaska Natives (AN), who have some of the highest smoking rates in the country. The study follows the Stage Model to first develop the Facebook intervention (Aim 1, Stage 1a) and then conduct a Stage 1b pilot randomized controlled trial (RCT) (Aim 2) to determine feasibility of the intervention vs. a quitline treatment referral. The significance is high as evidenced by preliminary data demonstrating acceptability of social media interventions by AN and promise of social media interventions for smoking cessation. However, one factor that undermines the premise is a lack of evidence discussed that AN respond to TIPS-like messaging by calling the quitline or otherwise seeking formal help to quit smoking. If this is an intended consequence of the web-based intervention, which will also use TIPS-type messaging, then there should be some AN-specific background information given to strengthen the scientific premise. This team of investigators has been productively working with AN and the Alaska Native Tribal Health Consortium (ANTHC) for several years. The plans for Stage 1A and Stage 1b are well described, including the inclusion of culturally-tailored aspects of the intervention. Overall the approach is very sound but there are several minor concerns. One aspect of the messaging that is not addressed in the Approach is rationale for casting messages as gain-framed or loss-framed; TIPS messages tend to be loss framed but there is evidence that smokers may be more responsive to gain-framed messages, especially if they have high reward sensitivity. If aims are completed and the preliminary efficacy trial demonstrates feasibility, the groundwork would be laid for a larger scale efficacy trial, which could have an overall high impact in the AN population. While the prevalence of smoking among AN is high, AN make up a relatively small proportion of all US smokers. However, knowledge gained from this research could be modified and transferred to other groups of Native Americans across the US for a larger overall impact.

1. Significance:

Strengths

- The scientific premise is strong: Investigators describe preliminary data demonstrating acceptability of social media interventions by AN and show that social media interventions are promising for smoking cessation.
- At the same time, more trials are needed, thus lending innovation to this line of research.

Weaknesses

- If the Facebook intervention is intended to prompt engagement in the quitline (as described in the application), and much of the Facebook intervention is modeled on TIPS, then it would be

helpful to know whether TIPS campaigns were successful in prompting this particular group of smokers (AN) to call the quitline. General statistics of success are quoted, but none are specific to AK or AN. The AK quitline and/or AK DOH may have some of this information through its own data collection program and the NAQC MDS.

- The following is a thought question that could use more discussion in the application to fortify significance. What is the evidence that AN need tailored smoking cessation interventions? The investigators describe a Community Advisory Board (CAB) experience in which AN members expressed preferences in seeing AN smokers (as opposed to non-AN smokers) tell their own stories. But is there published evidence that tailoring is necessary in this population? For example, an analogous disparity group is smokers who identify with LGBT. Literature on smoking cessation for these individuals is divided on whether there needs to be specific tailoring, as opposed to simply allocating more general evidence-based resources to this population in need.

2. Investigator(s):

Strengths

- This is a group of established investigators who have already been conducting NIH supported research with AN smokers and the ANTHC.

Weaknesses

- None noted.

3. Innovation:

Strengths

- There is a dearth of tailored interventions for AN and a dearth of trial data for interventions in this population.

Weaknesses

- None noted.

4. Approach:

Strengths

- A major strength is that the formative phase and trial phase of the study are scientifically rigorous and very well described.

Weaknesses

- There are several minor concerns.
- There is lack of detail around the control condition in the pilot randomized controlled trial (RCT). How will the eReferral be made—as a direct referral, or simply provision of the quitline number?
- Use of the quitline is also an intended outcome of the Facebook intervention. How will this be tracked? What level of detail will be gleaned? For example, ideal engagement would be calling the quitline, engaging in the maximum # of sessions, and using FDA approved medication offered by the quitline.
- As currently written, it seems treatment utilization (including use of quitline services) will be limited to self-report. Self-report is used widely but not ideal, and the approach would be more rigorous if it could be made objective. Objective verification could be obtained from the quitline

with permission from participants. Since they are providing informed consent already via Qualtrics, could this permission to receive quitline data be incorporated into the informed consent?

- For biochemical verification, what are the expected rates of return that this group believes it can achieve? Prior data suggest that biochemical verification attempted with a web-based sample has many potential barriers and limitations. (Cha et al, Addict Behav, 2017). For clinical trials, biochemical verification is necessary (Scheuermann et al, Addiction, 2017), yet for this type of research, alternative methods may be required.

5. Environment:

Strengths

- The existing partnerships that support this work, as well as the Mayo Clinic environment, which will handle the Facebook development, are well described and strong.

Weaknesses

- None noted.

Protections for Human Subjects:

Acceptable Risks and/or Adequate Protections

Data and Safety Monitoring Plan (Applicable for Clinical Trials Only):

Acceptable

- The plan for data safety is reasonable.

Inclusion of Women, Minorities and Children:

- Sex/Gender: Distribution justified scientifically
- Race/Ethnicity: Distribution justified scientifically
- For NIH-Defined Phase III trials, Plans for valid design and analysis: Not applicable
- Inclusion/Exclusion of Children under 18: Excluding ages <18; justified scientifically
- Individuals 19 and older are able to participate, which means that those who are 18 and younger are excluded. This is due to age 19 being the legal smoking age in AK.

Vertebrate Animals:

Not Applicable (No Vertebrate Animals)

Biohazards:

Not Applicable (No Biohazards)

Resource Sharing Plans:

Acceptable

Budget and Period of Support:

Recommend as Requested

Additional Comments to Applicant (Optional):

- What are the options for linking quitline referrals directly to the Facebook intervention? This could be achieved by having a link to the portal (if AK quitline has one) or having the moderator assist with these referrals. Similar scenario for smokefree.gov with texting or smartphone apps. How could referral to and use of these "intended consequences" of the intervention be more fully incorporated into the social media site?

CRITIQUE 3

Significance: 1
Investigator(s): 1
Innovation: 3
Approach: 4
Environment: 1

Overall Impact: Alaska Native persons have the highest prevalence of tobacco use among U.S. racial/ethnic groups, representing a high priority population for tobacco control. This R34 application proposes to develop a Facebook-based intervention targeted to Alaska Native daily smokers and to evaluate feasibility, utilization, and efficacy in a pilot randomized controlled trial. Significance is very high given that, if found to be effective, a culturally targeted social media intervention, combined with Alaska quitline services, that has state-wide reach would greatly improve utilization of evidence-based treatment and, in turn, reduce tobacco use. The scientific premise is strong with a balanced review of high quality research on tobacco use, treatment utilization, and quitting in Alaska Native people, cultural targeting of health communication messaging, and social media and tobacco treatment. The investigators and environment are exceptionally strong. Innovation is moderately high in that, although social media has been a popular channel for delivery of health behavior change intervention, there have been few studies of Facebook-based tobacco treatment and the proposed project would be the first to develop and pilot-test a culturally targeted intervention for Alaska Native smokers. The scientific rigor is strong, with numerous strengths but overall impact is reduced by several methodological weaknesses. These include: lack of stratification by cigarettes per day and reliance on participant reporting of quitline treatment utilization and engagement. Likelihood is high that the proposed developmental and pilot evaluation project will contribute valuable data to a future large-scale efficacy trial.

1. Significance:

Strengths

- Alaska Native persons have the highest prevalence of tobacco use among racial and ethnic groups. Quit attempt and successful quitting rates are low.
- Advances in knowledge about the potential for culturally targeted behavioral intervention to increase use of effective treatment and promote successful smoking cessation in this high priority disparity population.
- Social media offers a promising scalable strategy that addresses critical treatment access barriers to healthcare for Alaska Native people. These include geographic remoteness, climate, and travel costs.
- The scientific premise is strong, supported by rigorous theoretical, epidemiological, and experimental research on: tobacco use and in Alaska Native people, including cultural and

social factors that promote/interfere with quitting; social media and tobacco cessation treatment delivery; cultural targeting of health communication messages.

Weaknesses

- None noted.

2. Investigator(s):

Strengths

Principal Investigator Dr. Christi Patten is Professor of Psychology and Director of the Behavioral Health Research Program at the Mayo Clinic. She has impressive expertise in the development and evaluation of behavioral tobacco cessation treatments and community-based participatory research involving Native Americans.

- As a research nurse supervisor with the Alaska Native Tribal Health Consortium since 1998, Co-Investigator Dr. Kathryn Koller has extensive clinical and research experience in chronic disease risk reduction, including tobacco cessation, with the Alaska Native population.
- Well-regarded expertise in biostatistics (Co-Investigator Paul Decker, MS), use of social media for delivery of health behavior change interventions (Co-Investigator Dr. Judith Prochaska), tailored health communication, multi-media programs (Consultant Dr. Kenneth Resnicow).

Weaknesses

- None noted.

3. Innovation:

Strengths

- Culturally targeted Facebook intervention for Alaska Native smokers that has state-wide reach, designed to increase treatment utilization and promote smoking cessation.

Weaknesses

- Social media, including Facebook, has been used to deliver health behavior change interventions. Facebook-based tobacco interventions have been evaluated for general population smokers.

4. Approach:

Strengths

- Use of a community advisory committee to guide all project activities. An existing board was consulted as a part of the development of this R34 application.
- Well-conceived plan for the qualitative and quantitative phases to develop and refine the intervention content and the prototype including beta testing. Qualitative approach based in part on cultural variance and surface/deep structure frameworks (Resnicow et al).
- Intervention content to include a digital storytelling approach adapted from the CDC Tips from Former Smokers campaign. Closed and secret, moderated group structure and content organized to mimic a treatment manual.
- Inclusion and exclusion criteria are well-specified and justified.
- Strong recruitment outreach plan for the formative work and pilot trial.

- Assessment of the culturally-relevant value of interdependence (relationship orientation, collaborative efforts in lifestyle change) as one potential mediator of treatment effects.
- The 3-month intervention period duration is justified.
- Strong assessment plan that addresses feasibility, engagement/utilization (including use of non-study treatment), and preliminary efficacy.
- Biochemical verification of smoking abstinence at 1, 3, and 6 months. Remote collection of saliva cotinine for bioverification of abstinence (levels <15 ng/ml considered abstinent).

Weaknesses

- Important issues related to health literacy are not addressed.
- Lack of stratification by cigarettes smoked per day given the low (≥ 1/day in the past seven days) but appropriate threshold for study entry.
- Reliance on self-report of quitline treatment utilization.

5. Environment:

Strengths

- The Mayo Clinic (Rochester, MN) is especially well-suited for the proposed project. The Mayo Social Media Department, Clinical Office of Health Disparities Research, Office for Community Engagement in Research, and Native American Programs (Mayo Clinic Cancer Center) are particularly important assets.

Weaknesses

- None noted.

Protections for Human Subjects:

Acceptable Risks and/or Adequate Protections

- Minimal risk. Comprehensive protections.

Data and Safety Monitoring Plan (Applicable for Clinical Trials Only):

Acceptable

- o The DSMP is well done. A DSMB is planned.

Inclusion of Women, Minorities and Children:

- Sex/Gender: Distribution justified scientifically
- Race/Ethnicity: Distribution justified scientifically
- For NIH-Defined Phase III trials, Plans for valid design and analysis: Not applicable
- Inclusion/Exclusion of Children under 18: Excluding ages <18; justified scientifically
- Women are expected to comprise 50% of the sample. All participants will be racial minorities (Alaska Natives). Children will not be included. All justified scientifically.

Vertebrate Animals:

Not Applicable (No Vertebrate Animals)

Biohazards:

Not Applicable (No Biohazards)

Budget and Period of Support:

Recommend as Requested

THE FOLLOWING SECTIONS WERE PREPARED BY THE SCIENTIFIC REVIEW OFFICER TO SUMMARIZE THE OUTCOME OF DISCUSSIONS OF THE REVIEW COMMITTEE, OR REVIEWERS' WRITTEN CRITIQUES, ON THE FOLLOWING ISSUES:

PROTECTION OF HUMAN SUBJECTS: ACCEPTABLE

INCLUSION OF WOMEN PLAN: ACCEPTABLE

INCLUSION OF MINORITIES PLAN: ACCEPTABLE

INCLUSION OF CHILDREN PLAN: ACCEPTABLE

COMMITTEE BUDGET RECOMMENDATIONS: The budget was recommended as requested.

Footnotes for 1 R34 DA046008-01; PI Name: Patten, Christi A

NIH has modified its policy regarding the receipt of resubmissions (amended applications). See Guide Notice NOT-OD-14-074 at http://grants.nih.gov/grants/guide/notice-files/NOT-OD-14-074.html. The impact/priority score is calculated after discussion of an application by averaging the overall scores (1-9) given by all voting reviewers on the committee and multiplying by 10. The criterion scores are submitted prior to the meeting by the individual reviewers assigned to an application, and are not discussed specifically at the review meeting or calculated into the overall impact score. Some applications also receive a percentile ranking. For details on the review process, see http://grants.nih.gov/grants/peer_review_process.htm#scoring.

MEETING ROSTER

Interventions to Prevent and Treat Addictions Study Section
Risk, Prevention and Health Behavior Integrated Review Group
CENTER FOR SCIENTIFIC REVIEW
IPTA
10/12/2017

Notice of NIH Policy to All Applicants: Meeting rosters are provided for information purposes only. Applicant investigators and institutional officials must not communicate directly with study section members about an application before or after the review. Failure to observe this policy will create a serious breach of integrity in the peer review process, and may lead to actions outlined in NOT-OD-14-073 at https://grants.nih.gov/grants/guide/notice-files/NOT-OD-14-073.html and NOT-OD-15-106 at https://grants.nih.gov/grants/guide/notice-files/NOT-OD-15-106.html, including removal of the application from immediate review.

CHAIRPERSON(S)

SHOPTAW, STEVEN J, PHD
PROFESSOR
DEPARTMENT OF FAMILY MEDICINE
UNIVERSITY OF CALIFORNIA, LOS ANGELES
LOS ANGELES, CA 90024

MEMBERS

BERMAN, MITCHELL E, PHD *
PROFESSOR AND DEPARTMENT HEAD
DEPARTMENT OF PSYCHOLOGY
MISSISSIPPI STATE UNIVERSITY
MISSISSIPPI STATE, MS 39762

CROPSEY, KAREN L, PSYD *
PROFESSOR
DEPARTMENT OF PSYCHIATRY
UNIVERSITY OF ALABAMA AT BIRMINGHAM
BIRMINGHAM, AL 35294

DONOHUE, BRADLEY C, PHD *
PROFESSOR
DEPARTMENT OF PSYCHOLOGY
UNIVERSITY OF NEVADA, LAS VEGAS
LAS VEGAS, NV 89117

EDLUND, MARK J, PHD, MD *
SENIOR RESEARCH SCIENTIST
BEHAVIORAL HEALTH EPIDEMIOLOGY PROGRAM
RTI INTERNATIONAL
RESEARCH TRIANGLE PARK, NC 27709

FRIEDMANN, PETER D, MD, MPH
PROFESSOR
OFFICE OF RESEARCH
UNIVERSITY OF MASSACHUSETTS
MEDICAL SCHOOL - BAYSTATE
SPRINGFIELD, MA 01107

GRABOWSKI, JOHN G, PHD *
PROFESSOR
DEPARTMENT OF PSYCHIATRY
MEDICAL SCHOOL
UNIVERSITY OF MINNESOTA
MINNEAPOLIS, MN 55454

GRAY, KEVIN M, MD
PROFESSOR
DEPARTMENT OF PSYCHIATRY
AND BEHAVIORAL SCIENCES
MEDICAL UNIVERSITY OF SOUTH CAROLINA
CHARLESTON, SC 29425

HARTZLER, BRYAN J, PHD *
RESEARCH SCIENTIST
ALCOHOL AND DRUG ABUSE INSTITUTE
UNIVERSITY OF WASHINGTON
SEATTLE, WA 98105-4631

HEIL, SARAH H, PHD
ASSOCIATE PROFESSOR
DEPARTMENT OF PSYCHIATRY
COLLEGE OF MEDICINE
UNIVERSITY OF VERMONT
BURLINGTON, VT 05401

HITSMAN, BRIAN L, PHD
ASSOCIATE PROFESSOR
DEPARTMENT OF PREVENTIVE MEDICINE
FEINBERG SCHOOL OF MEDICINE
NORTHWESTERN UNIVERSITY
CHICAGO, IL 60611

MAHABEE-GITTENS, E. MELINDA, MD
PROFESSOR
DIVISION OF EMERGENCY MEDICINE
CINCINNATI CHILDREN'S HOSPITAL MEDICAL CENTER
CINCINNATI, OH 45229

MARTIN, LAURA E, PHD *
ASSOCIATE PROFESSOR
DEPARTMENT OF PREVENTIVE MEDICINE AND
PUBLIC HEALTH, HOGLUND BRAIN IMAGING CENTER
UNIVERSITY OF KANSAS MEDICAL CENTER
KANSAS CITY, KS 66160

MCGOVERN, MARK P, PHD
PROFESSOR
DEPARTMENT OF PSYCHIATRY
AND BEHAVIORAL SCIENCES
STANFORD UNIVERSITY
PALO ALTO, CA 93404

MCKAY, JAMES R, PHD
PROFESSOR
DEPARTMENT OF PSYCHIATRY
UNIVERSITY OF PENNSYLVANIA
PHILADELPHIA, PA 19104

MENDELSON, TAMAR, PHD *
ASSOCIATE PROFESSOR
DEPARTMENT OF MENTAL HEALTH
JOHNS HOPKINS BLOOMBERG SCHOOL OF PUBLIC HEALTH
BALTIMORE, MD 21205

OKUYEMI, KOLAWOLE S MD, MPH
PROFESSOR AND DEPARTMENT CHAIR
DEPARTMENT OF FAMILY AND PREVENTIVE MEDICINE
UNIVERSITY OF UTAH SCHOOL OF MEDICINE
SALT LAKE CITY, UT 84108

OLIVETO, ALISON, PHD
PROFESSOR AND VICE CHAIR FOR RESEARCH
DEPARTMENT OF PSYCHIATRY
UNIVERSITY OF ARKANSAS FOR MEDICAL SCIENCES
LITTLE ROCK, AR 72205

OSILLA, KAREN C, PHD
SENIOR BEHAVIORAL SCIENTIST
RAND CORPORATION
SANTA MONICA, CA 90404

STOOPS, WILLIAM W, PHD
PROFESSOR
DEPARTMENT OF BEHAVIORAL SCIENCE
UNIVERSITY OF KENTUCKY
LEXINGTON, KY 40536

TAXMAN, FAYE S, PHD
UNIVERSITY PROFESSOR
DEPARTMENT OF CRIMINOLOGY,
LAW AND SOCIETY
GEORGE MASON UNIVERSITY
FAIRFAX, VA 22020

TINDLE, HILARY A, MD, MPH
ASSOCIATE PROFESSOR
DIVISION OF INTERNAL MEDICINE
VANDERBILT UNIVERSITY MEDICAL CENTER
NASHVILLE, TN 37203

TSOH, JANICE Y, PHD
PROFESSOR
DEPARTMENT OF PSYCHIATRY
LANGLEY PORTER PSYCHIATRIC INSTITUTE
UNIVERSITY OF CALIFORNIA SAN FRANCISCO
SAN FRANCISCO, CA 94143

VELASQUEZ, MARY M, PHD
CENTENNIAL PROFESSOR AND DIRECTOR
HEALTH BEHAVIOR RESEARCH
AND TRAINING INSTITUTE
SCHOOL OF SOCIAL WORK
UNIVERSITY OF TEXAS AT AUSTIN
AUSTIN, TX 78712

WACHHOLTZ, AMY B, PHD *
ASSISTANT PROFESSOR
DEPARTMENT OF PSYCHOLOGY
UNIVERSITY OF COLORADO DENVER
DENVER, CO 80217

WALTON, MAUREEN A, PHD, MPH
PROFESSOR
DEPARTMENT OF PSYCHIATRY
UNIVERSITY OF MICHIGAN
ANN ARBOR, MI 48105

WORLEY, MATTHEW J, PHD *
ASSISTANT PROFESSOR
DEPARTMENT OF PSYCHIATRY
UNIVERSITY OF CALIFORNIA, SAN DIEGO
LA JOLLA, CA 92093

SCIENTIFIC REVIEW OFFICER

MINTZER, MIRIAM, PHD
SCIENTIFIC REVIEW OFFICER
CENTER FOR SCIENTIFIC REVIEW
NATIONAL INSTITUTES OF HEALTH
BETHESDA, MD 20892

EXTRAMURAL SUPPORT ASSISTANT

WATTS, MELISSA D
EXTRAMURAL SUPPORT ASSISTANT
CENTER FOR SCIENTIFIC REVIEW
NATIONAL INSTITUTE FOR HEALTH
BETHESDA, MD 20892

* Temporary Member. For grant applications, temporary members may participate in the entire meeting or may review only selected applications as needed.

Consultants are required to absent themselves from the room during the review of any application if their presence would constitute or appear to constitute a conflict of interest.
